# Assembly and comparative analysis of the complete mitochondrial genome of *Trigonella foenum-graecum* L.

**DOI:** 10.1186/s12864-023-09865-6

**Published:** 2023-12-08

**Authors:** Yanfeng He, Wenya Liu, Jiuli Wang

**Affiliations:** 1 College of Pharmacy, Qinghai Minzu University, Xining, 810007 Qinghai China; 2 The College of Ecological Environment and Resources, Qinghai Minzu University, Xining, 810007 Qinghai China

**Keywords:** *Trigonella foenum-graecum* L., Mitochondrial genome, Sequence characteristics, Phylogenetic analysis

## Abstract

**Background:**

*Trigonella foenum-graecum* L. is a Leguminosae plant, and the stems, leaves, and seeds of this plant are rich in chemical components that are of high research value. The chloroplast (cp) genome of *T. foenum-graecum* has been reported, but the mitochondrial (mt) genome remains unexplored.

**Results:**

In this study, we used second- and third-generation sequencing methods, which have the dual advantage of combining high accuracy and longer read length. The results showed that the mt genome of *T. foenum-graecum* was 345,604 bp in length and 45.28% in GC content. There were 59 genes, including: 33 protein-coding genes (PCGs), 21 tRNA genes, 4 rRNA genes and 1 pseudo gene. Among them, 11 genes contained introns. The mt genome codons of *T. foenum-graecum* had a significant A/T preference. A total of 202 dispersed repetitive sequences, 96 simple repetitive sequences (SSRs) and 19 tandem repetitive sequences were detected. Nucleotide diversity (Pi) analysis counted the variation in each gene, with *atp6* being the most notable. Both synteny and phylogenetic analyses showed close genetic relationship among *Trifolium pratense*, *Trifolium meduseum*, *Trifolium grandiflorum*, *Trifolium aureum*, *Medicago truncatula* and *T. foenum-graecum*. Notably, in the phylogenetic tree, *Medicago truncatula* demonstrated the highest level of genetic relatedness to *T. foenum-graecum*, with a strong support value of 100%. The interspecies non-synonymous substitutions (Ka)/synonymous substitutions (Ks) results showed that 23 PCGs had Ka/Ks < 1, indicating that these genes would continue to evolve under purifying selection pressure. In addition, setting the similarity at 70%, 23 homologous sequences were found in the mt genome of *T. foenum-graecum.*

**Conclusions:**

This study explores the mt genome sequence information of *T. foenum-graecum* and complements our knowledge of the phylogenetic diversity of Leguminosae plants.

## Background

*T. foenum-graecum* is a representative of family Leguminosae, which has a long history [1]. Its leaves are used as a vegetable and the seeds are used to make spices [1]. In addition, *T. foenum-graecum* is rich in sugars, proteins, lipids, and other nutrients [2]; the seeds and leaves contain a variety of active chemicals, such as flavonoids and alkaloids [3]which are used in a variety of ways due to their analgesic and anti-inflammatory, antioxidant, and hypoglycaemic activity [4]. The composition, structure and utility of *T. foenum-graecum* have been intensively studied in the literature, but the mt genome has not been sequenced so far.

Four major elements could be distinguished in the structure of mitochondria: matrix, inner and outer membrane, and intermembrane space [5]. Mitochondria possess their own genome (mt genome), and mt genome encodes some RNAs and polypeptides [6]. Mitochondria are one of the energy converters in plant cells. In addition to providing energy, it can also serve as a hub for metabolism or signaling, and is closely related to apoptosis, necrosis, differentiation and other vital activities [7, 8]. It plays an important role in the growth and development of plants [9, 10].

The size, structure and gene content of mt genome vary greatly, but the number of functional genes does not change much, showing complex and relatively conservative characteristics [11, 12]. It is generally accepted that plant mtDNA consists of a “master circle” conformation of the entire sequence content of the genome and a set of subgenomic circles generated by repeat-mediated recombination [13]. Because of this, “master circle” and subgenomic circles can coexist in the cell, making the structure of plant mt genomes more complex and difficult to study. The mt genomes of angiosperms usually range from 200 to 750 kb [12], and the size varies significantly among plants. The number of editing sites for mt RNA in higher plants is greater than 400, which is about 13 times the number of editing sites for cp RNA [14]. Repeated sequences of plant mt genomes undergo frequent recombination, making their structures increasingly complex [15]. Based on the above multiple factors, sequence characterization and phylogenetic analysis in the mt genome of *T. foenum-graecum* were investigated in depth for a more comprehensive understanding of the genetic characteristics and phylogenetic relationships of genus *Trigonella* (Leguminosae).

## Results

### Basic characteristics of *T. foenum-graecum* mt genome

**Fig. 1:**
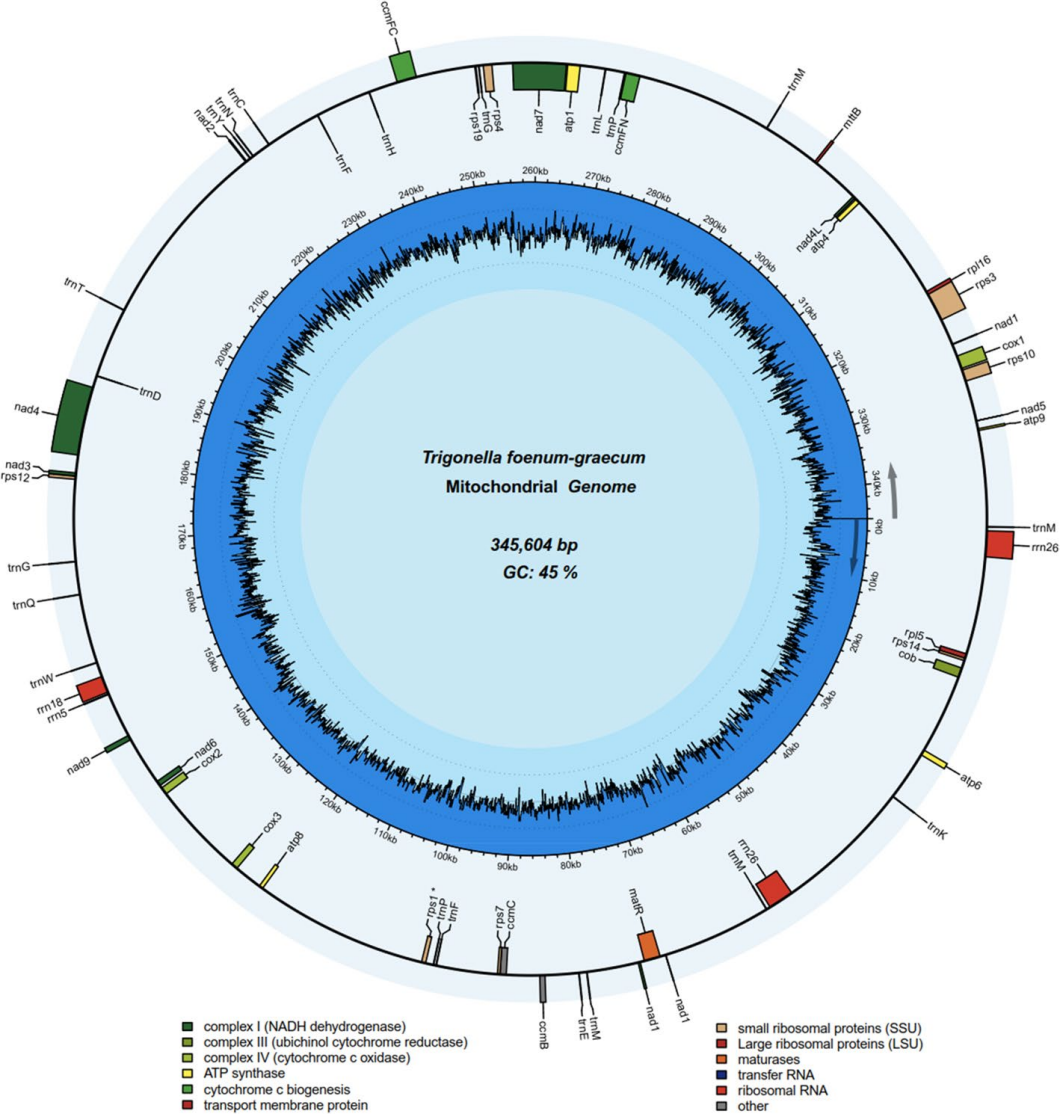
The circular map of the *T. foenum-graecum* mt genome. The different colors represent genes belonging to different functional groups

The *T. foenum-graecum* mt genome is circular in structure with a total length of 345,604 bp and GC content of 45.28%. The GC content of PCGs (42.72%) is lower than that of tRNA (52.41%) and rRNA (51.2%). The mt genome structure is shown in Fig. 1. There are 59 genes, including 33 PCGs, 21 tRNA genes, 4 rRNA genes and 1 pseudo gene. The classification of genes in the mt genome of *T. foenum-graecum* is shown in Table 1. Among them, there are 11 genes with introns (*ccmFC*, *nad1*, *nad2*, *nad4*, *nad5*, *nad7*, *rps3*, *rps7*, *rps10*, *trnP-CGG*, *trnT-TGT*) containing a total of 25 introns. Genes encoding subunits of NADH dehydrogenase contain the largest number of introns, 19 in total. In addition, two copies of *rrn26*, *trnF-GAA*, *trnG-GCC* and four copies of *trnM-CAT* were found in the *T. foenum-graecum* mt genome. The *rps1* is a pseudo gene.

**Table 1:** Gene classification table

Group of genes	Gene name	Length	Start codon	Stop codon	Amino acid
ATP synthase	*atp1*	1518	ATG	TAA	506
	*atp4*	588	ATG	TAA	196
	*atp6*	663	ATG	CAA (TAA)	221
	*atp8*	483	ATG	TAA	161
	*atp9*	225	ATG	TAA	75
Cytochrome c biogenesis	*ccmB*	621	ATG	TGA	207
	*ccmC*	747	ATG	TGA	249
	ccmFC*	1431	ATG	TAG	477
	*ccmFN*	1728	ATG	TGA	576
Ubichinol cytochrome c reductase	*cob*	1179	ATG	TGA	393
Cytochrome c oxidase	*cox1*	1584	ATG	TAA	528
	*cox2*	906	ATG	TAA	302
	*cox3*	798	ATG	TGA	266
Maturases	*matR*	1986	ATG	TAG	662
Transport membrane protein	*mttB*	312	ATG	TGA	104
NADH dehydrogenase	nad1****	978	ACG (ATG)	TAA	326
	nad2****	1467	ATG	TAA	489
	*nad3*	357	ATG	TAA	119
	nad4***	1488	ATG	TGA	496
	*nad4L*	303	ACG (ATG)	TAA	101
	nad5****	2010	ATG	TAA	670
	*nad6*	618	ATG	TAA	206
	nad7****	1185	ATG	TAG	395
	*nad9*	591	ATG	TAA	197
Large subunit ribosomal proteins (LSU)	*rpl16*	558	ATG	TAA	186
	*rpl5*	564	ATG	TAA	188
Small subunit ribosomal proteins (SSU)	rps10*	411	ATG	TAA	137
	*rps12*	372	ATG	TGA	124
	*rps14*	303	ATG	TAG	101
	*rps19*	138	ATG	TGA	46
	rps3*	1677	ATG	TAA	559
	*rps4*	1050	ATG	TAA	350
	rps7*	276	ATG	TAA	92
Ribosomal RNAs	*rrn18*	2008			
	*rrn26(2)*	(3137,3137)			
	*rrn5*	115			
Transfer RNAs	*trnC-GCA*	71			
	*trnD-GTC*	74			
	*trnE-TTC*	72			
	*trnF-GAA(2)*	(64,74)			
	*trnG-GCC(2)*	(72,72)			
	*trnH-GTG*	74			
	*trnK-TTT*	73			
	*trnL-CAA*	82			
	*trnM-CAT(4)*	(73,74,74,74)			
	*trnN-GTT*	72			
	trnP-CGG*	83			
	*trnP-TGG*	75			
	*trnQ-TTG*	72			
	trnT-TGT*	75			
	*trnW-CCA*	74			
	*trnY-GTA*	83			
Pseudo gene	*rps1*	514	ATG	–	171

The digit in brackets after the gene names indicate the number of gene copies. The number of asterisks (*) after the gene name represents the number of introns identified within a particular gene

In protein-coding genes, the most frequent start and stop codons are ATG and TAA, respectively. The 21 tRNA genes that encode 15 out of 20 amino acids, including: methionine (Met), lysine (Lys), glutamate (Glu), phenylalanine (Phe), proline (Pro), tryptophan (Trp), glutamine (Gln), glycine (Gly), aspartate (Asp), threonine (Thr), tyrosine (Tyr), asparagine (Asn), cysteine (Cys), histidine (His), and leucine (Leu). It shows that some amino acids were encoded by two tRNA genes with different anticodons (*trnP-CGG* and *trnP-TGG*) but there are also tRNA genes that are duplicated (*trnF-GAA* and *trnG-GCC*) or present in higher number (4) of copies (*trnM-CAT*).

### Prediction of RNA editing sites

**Fig. 2:**
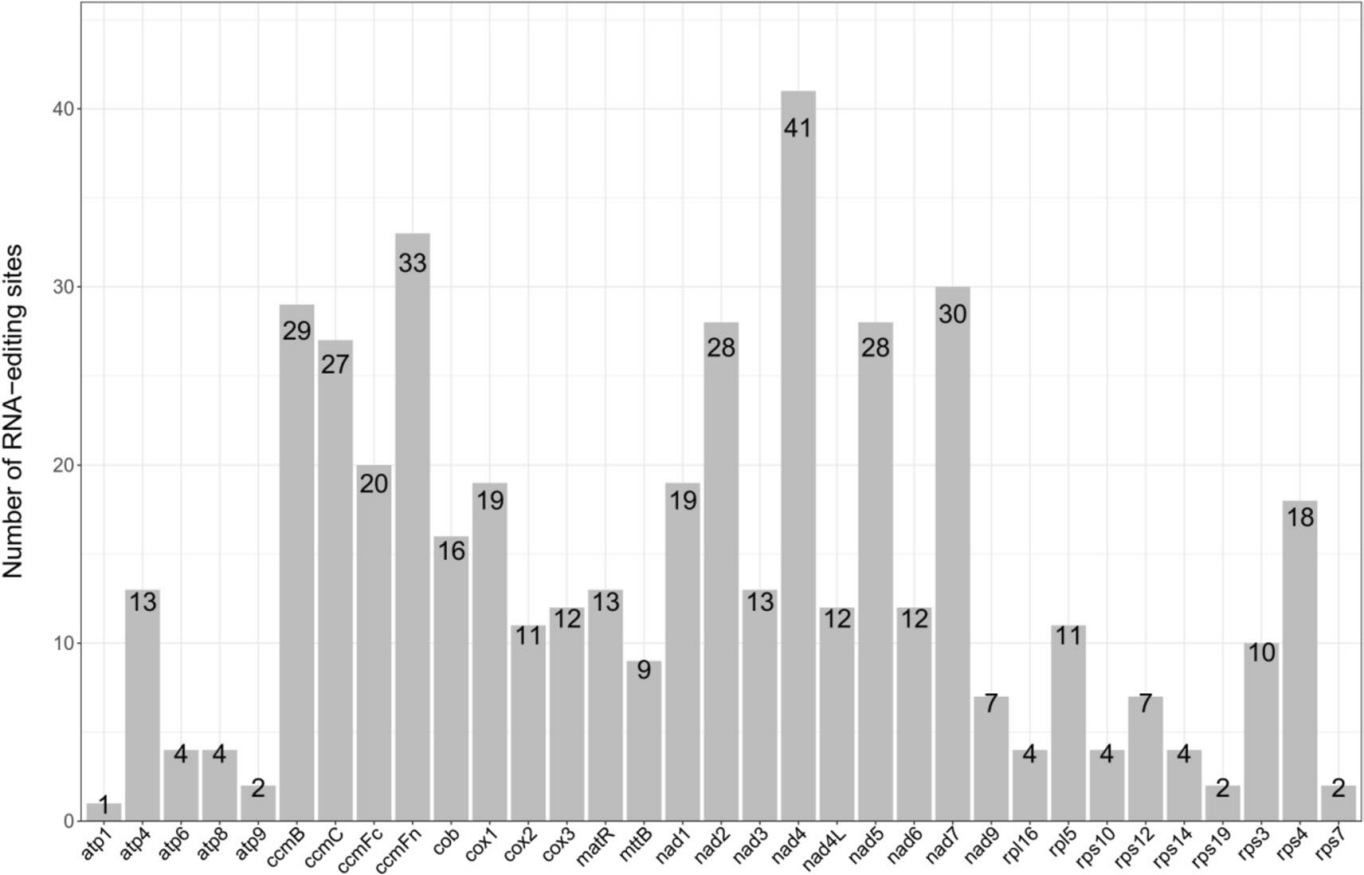
Distribution of RNA editing sites. The horizontal coordinate represents the gene name and the vertical coordinate represents the number of RNA edits

RNA editing affects gene expression and RNA stability through base substitution, insertion or deletion and plays an important role in promoting transcriptional diversity and enriching the variety of proteins [16, 17]. RNA editing sites were predicted for the mt genome of *T. foenum-graecum*, and a total of 465 RNA editing sites were predicted in 33 PCGs, and all RNA editing sites were of C to T conversions. The number of RNA editing sites in particular genes is shown in Fig. 2. Genes encoding ATP synthase (except *atp4*), transport membrane protein, maturase and ribosomal proteins (except *rpl5*, *rps4*, *rps3*) were found to have a relatively low number of RNA editing-derived substitutions (1–10 editing sites), while genes associated with cytochrome c biogenesis, ubichinol cytochrome c reductase, cytochrome c oxidase, and NADH dehydrogenase subunits (except *nad9*) were intensively edited (10–41 editing sites). Among them, *nad4* has the highest number of RNA editing sites.

The RNA editing sites were classified according to the hydrophilicity of amino acids, as shown in Table 2. It includes five types of edits: hydrophilic-hydrophilic, hydrophobic-hydrophobic, hydrophilic-hydrophobic, hydrophobic-hydrophilic and hydrophilic-stop. Among them, 13.12% of the amino acids remains hydrophilic; 31.83% of the amino acids remains hydrophobic; 47.53% of the amino acids changes from hydrophilic to hydrophobic; 6.45% of the amino acids changes from hydrophobic to hydrophilic; and 1.08% of the amino acids are prematurely terminated as a result of the distortion of the encoded information. Premature termination occurs in *atp6*, *ccmFc*, and *cox1* in the *T. foenum-graecum* mt genome. In addition, a total of 32 codon transitions are involved, with conversions TCA (S) to TTA (L) being the most common, with 68 editing sites.

**Table 2:** Classification table of RNA editing sites

Type	RNA-editing	Number	Percentage
hydrophilic-hydrophilic	CAC (H)= > TAC (Y)	9	13.12%
	CAT (H)= > TAT (Y)	14	
	CGC (R)= > TGC (C)	11	
	CGT (R)= > TGT (C)	27	
	total	61	
hydrophobic-hydrophobic	CCA (P)= > CTA (L)	39	31.83%
	CCC (P)= > CTC (L)	12	
	CCC (P)= > TTC (F)	6	
	CCG (P)= > CTG (L)	28	
	CCT (P)= > CTT (L)	23	
	CCT (P)= > TTT (F)	10	
	CTC (L)= > TTC (F)	7	
	CTT (L)= > TTT (F)	14	
	GCA (A)= > GTA (V)	1	
	GCC (A)= > GTC (V)	1	
	GCG (A)= > GTG (V)	4	
	GCT (A)= > GTT (V)	3	
	total	148	
hydrophilic-hydrophobic	ACA (T)= > ATA (I)	5	47.53%
	ACC (T)= > ATC (I)	1	
	ACG (T)= > ATG (M)	7	
	ACT (T)= > ATT (I)	3	
	CGG (R)= > TGG (W)	34	
	TCA (S)= > TTA (L)	68	
	TCC (S)= > TTC (F)	25	
	TCG (S)= > TTG (L)	40	
	TCT (S)= > TTT (F)	38	
	total	221	
hydrophobic-hydrophilic	CCA (P)= > TCA (S)	4	6.45%
	CCC (P)= > TCC (S)	7	
	CCG (P)= > TCG (S)	3	
	CCT (P)= > TCT (S)	16	
	total	30	
hydrophilic-stop	CAA (Q)= > TAA (X)	1	1.08%
	CAG (Q)= > TAG (X)	2	
	CGA (R)= > TGA (X)	2	
	total	5	

Detailed analysis of RNA editing sites revealed that 151 (32.47%) of these sites were located on the first base of the triplet codon and 298 (64.09%) on the second base of the codon. In addition we observed that the first and second bases of the same codon could be also edited and then amino acid changed from the original proline (CCT) to phenylalanine (TTT). In the study it was also found that the highest number of leucine was present after RNA editing. This includes: 108 sites converted from serine to leucine and 102 sites converted from proline to leucine.

### Codon preference

**Fig. 3:**
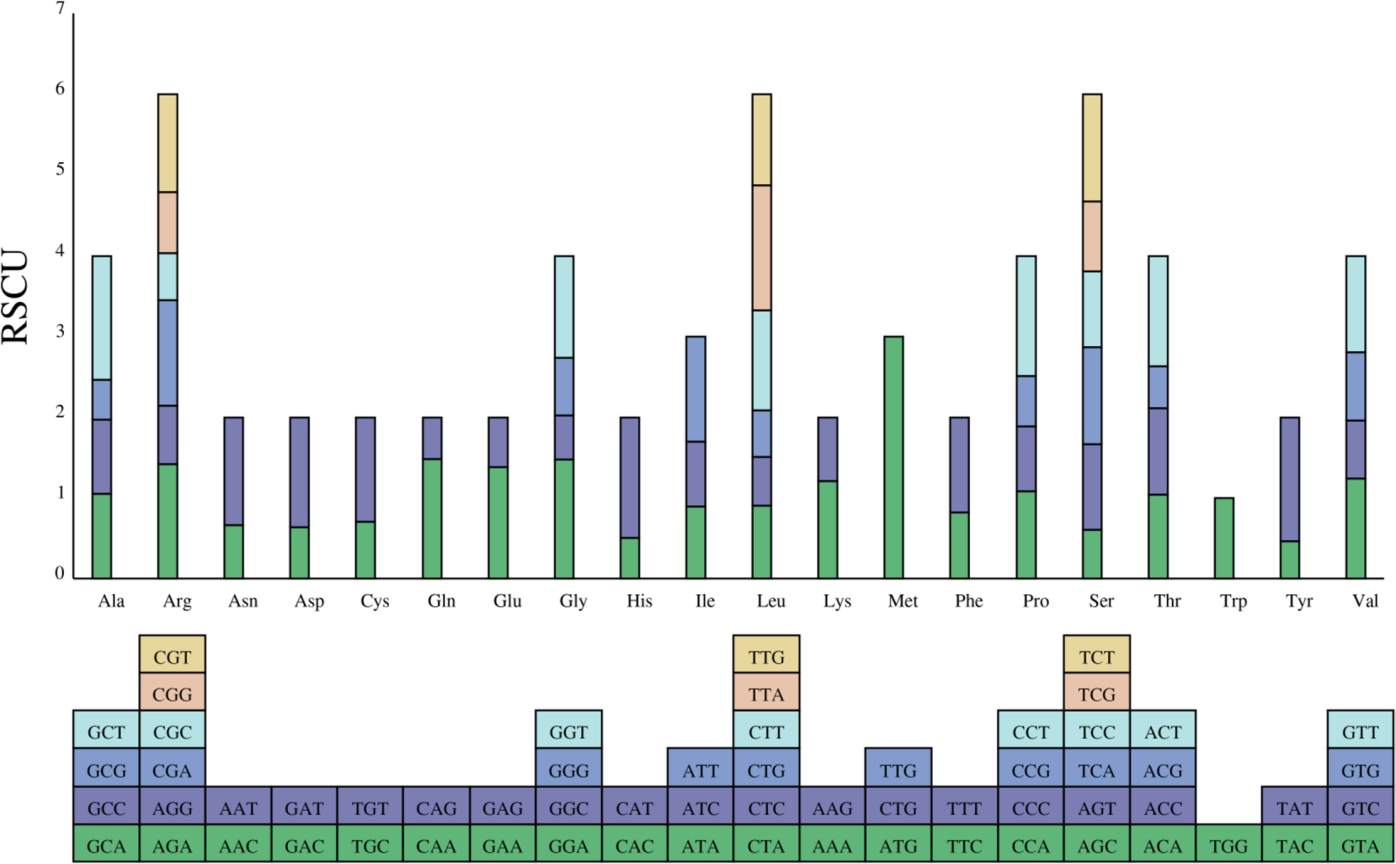
Relative synonymous codon usage (RSCU) histogram. The square below represents all codons encoding each amino acid and the height of the bar above represents the sum of all codon RSCU values

The study in mt genome codon preference of *T. foenum-graecum* showed that 32 codons have relative synonymous codon usage (RSCU) > 1, of which 29 ended with A or T, accounting for 90.63%. In addition, the 96 bases that make up the 32 codons contain 30 A and 32 T bases, indicating that codons with preference use more A/T bases in their composition. Thus, the *T. foenum-graecum* mt genome has a significant AT preference. The schematic diagram of codon preference is shown in Fig. 3.

### Repeated sequences

Dispersed repetitive sequences are repetitive units that are present in a scattered form throughout the genome [18]. A total of 202 dispersed repeat sequences were detected in the *T. foenum-graecum* mt genome, including 108 forward repeats (F) and 94 palindrome repeats (P), with repeat lengths mostly concentrated between 30 and 60 (83). The total length of the dispersed repetitive sequences is 47,506 bp, accounting for 13.75% of the total length of the mt genome. The length of each repeat sequence and the number of repeat types are detailed in Table 3.

**Table 3:** Distribution of dispersed repeat sequences

Length	Dispersed type	Number
20–29	P	2
	F	2
30–39	P	16
	F	19
40–49	P	14
	F	9
50–59	P	11
	F	14
60–69	P	2
	F	3
70–79	P	5
	F	11
80–89	P	3
	F	1
90–99	P	9
	F	9
100–199	P	18
	F	31
≥200	P	14
	F	9

*P* Palindrome repeats, *F *Forward repeats

**Fig. 4:**
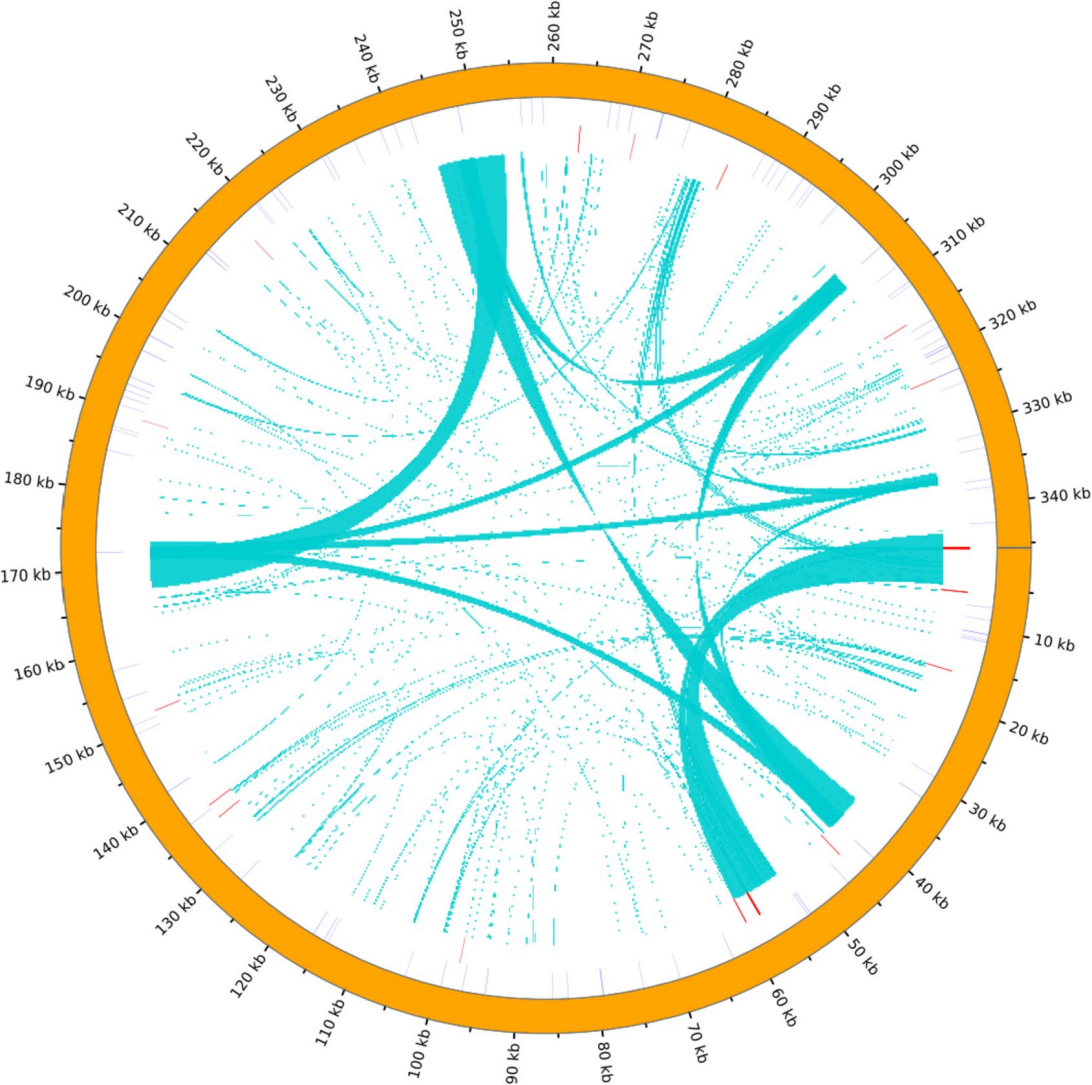
Distribution of repetitive sequences within the *T. foenum-graecum* mt genome. The outermost circle is the SSRs, followed by the tandem repeat sequences, and the innermost concatenation is the dispersed repeat sequences

Tandem repeat are core repeating units of 1 to 200 bases repeated several times in tandem and are widely present in eukaryotic and some prokaryotic genomes [18, 19]. A total of 19 tandem repeats were detected in the *T. foenum-graecum* mt genome, with length distributions ranging from 5 to 57, and 13 tandem repeats had a match rate of > 97%, as shown in Table 4. The distribution of repetitive sequences on the genome is shown in Fig. 4.

**Table 4:** Distribution of tandem repeat sequences

NO.	Size	Repeat sequence	Percent Matches
1	36	TAACATAGACCCTCTTTACTTACAGTCGAGCTCTAT	98
2	57	ATATGAAGTTCTAATATTATCTGCACTAAGAAGTGATTACGACTTGTTGTAGATGA	89
3	32	GAGAGGTATGAAAGCGATACTCGACTGATAAG	82
4	22	TTCGATGTAATTGATTTCGCCA	100
5	36	AGGGTCTATGTTAATAGAGCTCGACTGTAAGTAAAG	100
6	30	CGGAGGTTGAGGAGGAGTTTCGGGCTGCTG	64
7	16	CTTGTTATTAGTAAAG	100
8	27	TCTGTATCACTTCTTTACTTGGCTTAT	100
9	27	ATTCTCAATCCACGACGACTATTAACG	100
10	25	TTGATGAACAAGAAGGAACGAAGTG	100
11	12	ATTTATAGCAGC	100
12	15	TCTGACGTCCTTCCT	100
13	19	AATTATCTTATCTAAAATA	70
14	19	CACCTGCAGTTTGGTGCAG	88
15	28	TGCAGGCGAATAGAAAGAGCCCGGCACC	100
16	25	GGGTGAGGGATTAATAAACTAGCTC	100
17	5	ATTCA	100
18	9	GAGACTTTTG	90
19	36	CTTTACTTACAGTCGAGCTCTATTAACATAGACCCT	100

SSRs are composed of tandemly repeated DNA motifs (1–6 nt) with the advantages of high variability, reproducibility, multiallelic nature, relative abundance and good genome coverage, which are resources for the development of polymorphic DNA markers and can be widely used in plant genetic breeding [20–23]. A total of 96 SSRs were detected in the *T. foenum-graecum* mt genome, including 11 monomers, 21 dimers, 10 trimers, 34 tetramers, 16 pentamers and 4 hexamers. Among them, tetramers are the most abundant type of repeats, accounting for 35.42% of the total SSRs, whereas hexamers are the least frequent repeat type, accounting for only 4.17% of the total SSRs. Basic characteristics of identified SSRs is shown in Table 5.

**Table 5:** Distribution of SSRs

SSR type	Repeats	total
monomer	A/T	10
	C/G	1
dimer	AC/GT	1
	AG/CT	15
	AT/AT	5
trimer	AAC/GTT	1
	AAG/CTT	4
	AAT/ATT	4
	ATC/ATG	1
tetramer	AAAC/GTTT	2
	AAAG/CTTT	8
	AAAT/ATTT	3
	AACC/GGTT	1
	AAGC/CTTG	3
	AAGT/ACTT	3
	AATG/ATTC	3
	AATT/AATT	1
	ACAG/CTGT	1
	ACAT/ATGT	1
	ACCG/CGGT	1
	ACGG/CCGT	2
	ACTG/AGTC	1
	AGCC/CTGG	1
	AGCT/AGCT	1
	AGGC/CCTG	1
	CCCG/CGGG	1
pentamer	AAAAG/CTTTT	1
	AAAAT/ATTTT	1
	AAACC/GGTTT	4
	AAACT/AGTTT	1
	AAATT/AATTT	1
	AACTG/AGTTC	1
	AACTT/AAGTT	2
	AAGAT/ATCTT	1
	AAGCT/AGCTT	1
	AATTC/AATTG	1
	ACACC/GGTGT	1
	ACGGC/CCGTG	1
hexamer	AAACTT/AAGTTT	2
	AAATGG/ATTTCC	1
	AGATAT/ATATCT	1

### Nucleotide diversity

**Fig. 5:**
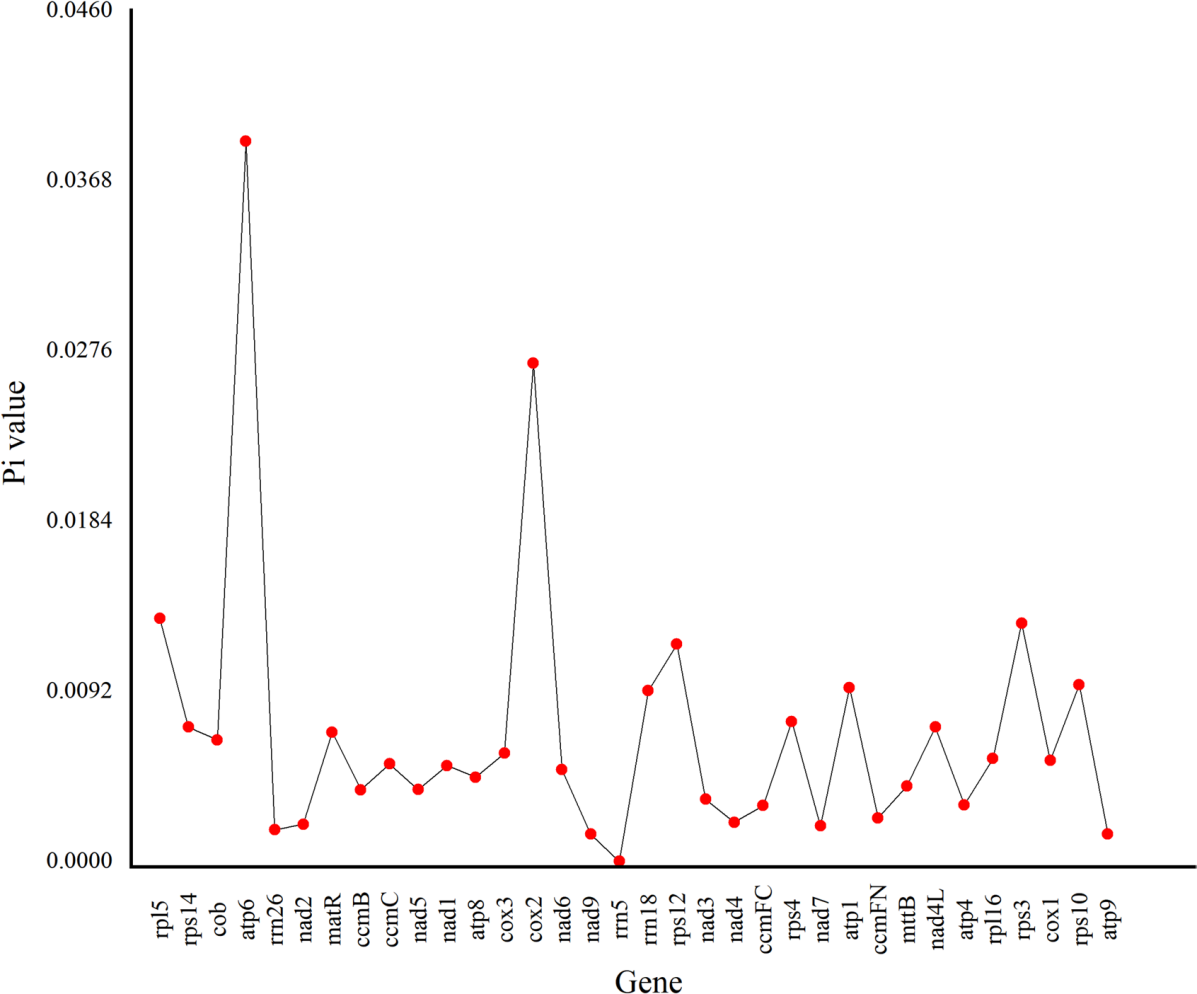
Nucleotide diversity (Pi) values for 34 mt genes shared by *T. foenum-graecum*, *Trifolium pratense*, *Trifolium meduseum*, *Trifolium grandiflorum*, *Trifolium aureum* and *Medicago truncatula*

Nucleotide diversity (Pi) ranged from 0 to 0.03891. The highest Pi value (0.03891) was observed for *atp6*. High nucleotide diversity was also observed for *rps12*, *rps3*, *rpl5*, and *cox2*, which were 0.01174, 0.01288, 0.01314 and 0.02692, respectively. Pi values of each gene are shown in Fig. 5.

### Synteny and phylogenetic analysis

**Fig. 6:**
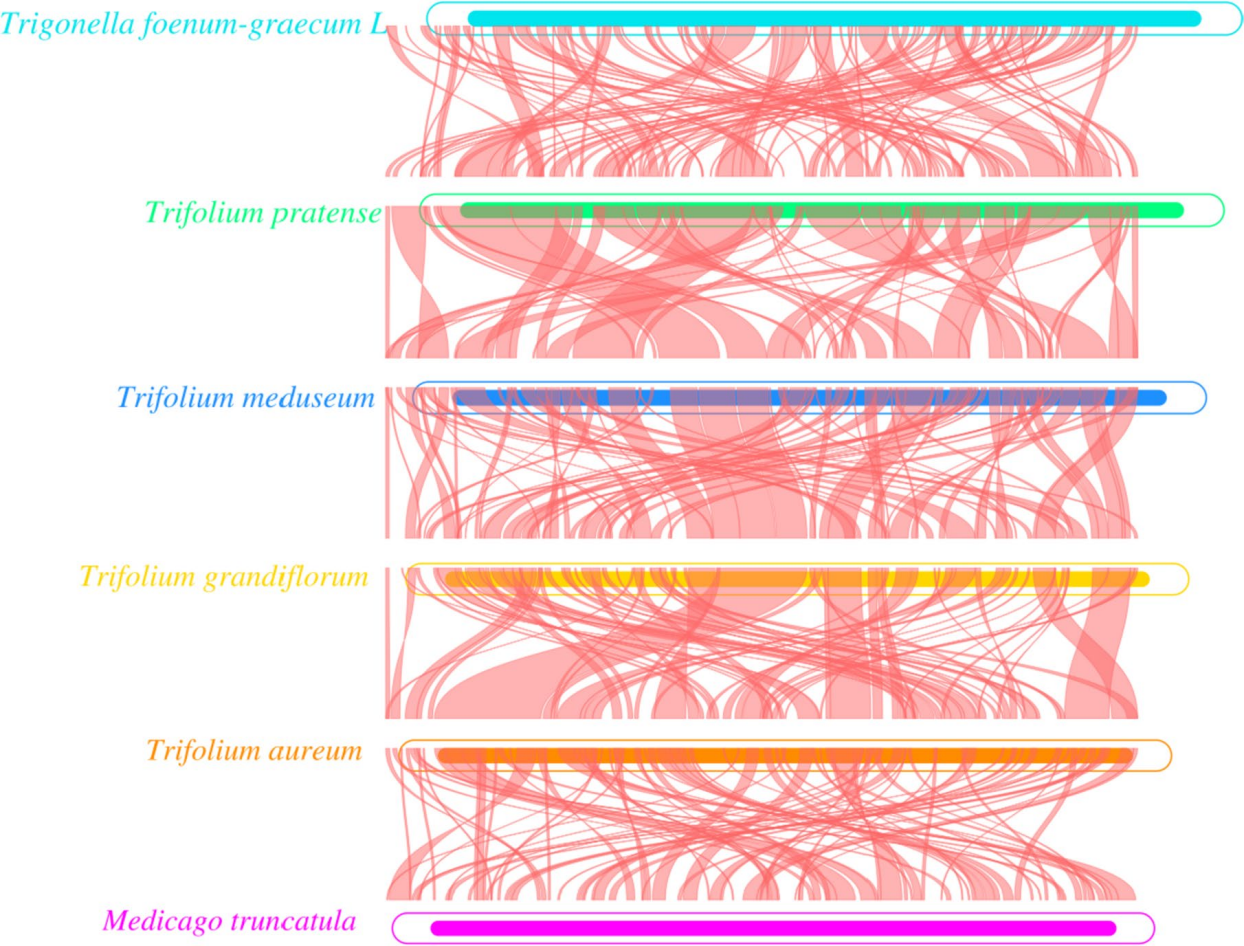
Mt genome sequence synteny analysis. The boxes in each row indicate a genome, and the connecting lines in the middle indicate homologous regions

**Fig. 7:**
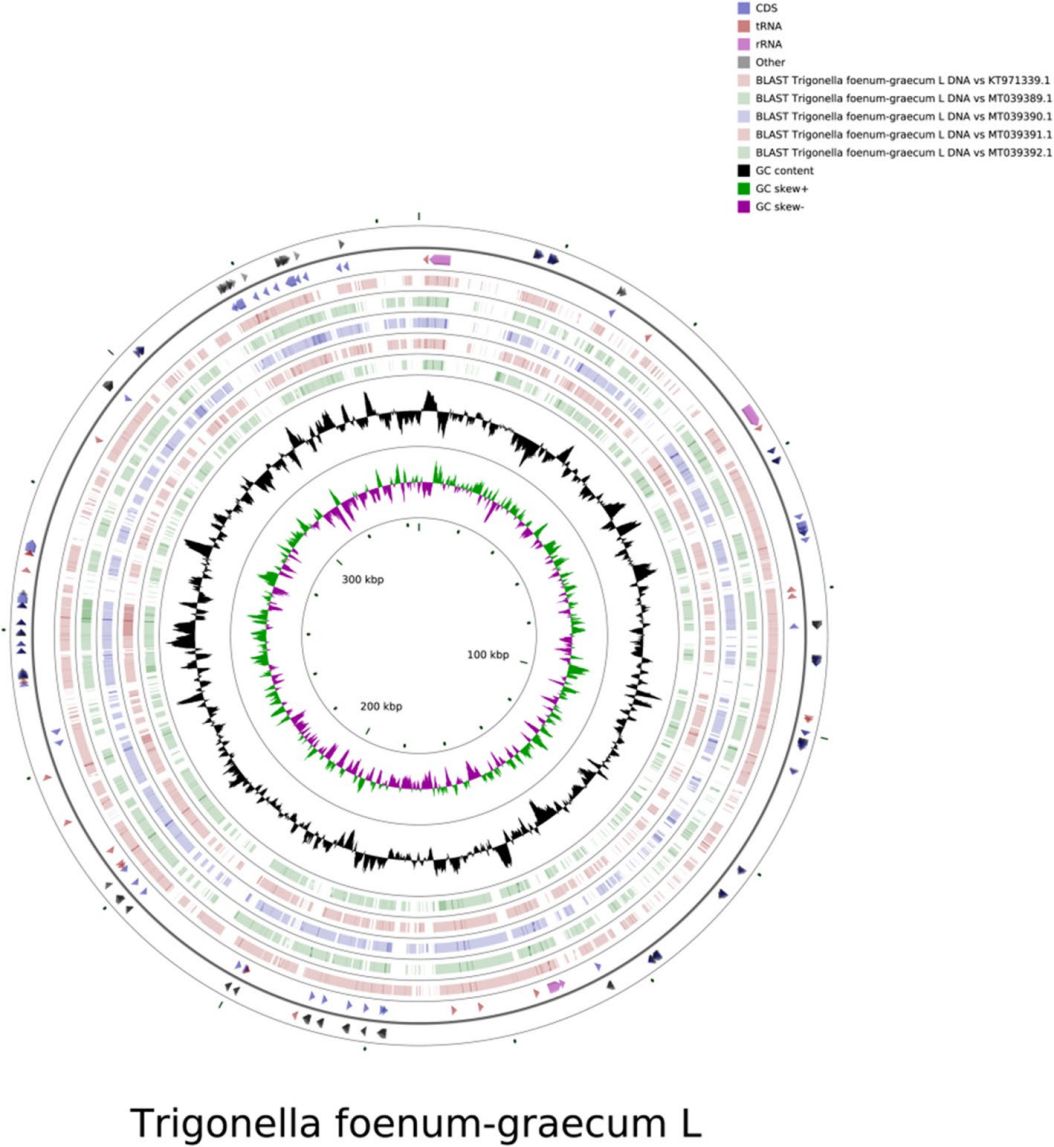
Comparison of the mt genomes structures of *T. foenum-graecum* (OP605625) and *Medicago truncatula* (KT971339.1), *Trifolium pratense* (MT039389.1), *Trifolium meduseum* (MT039390.1), *Trifolium grandiflorum* (MT039391.1), *Trifolium aureum* (MT039392.1). The two outermost circles depict the gene length and their orientation within the genome; subsequent circles represent the results of the comparison of *T. foenum-graecum* mitogenome with other reference genomes i.e.: the third circle: *T. foenum-graecum* vs KT971339.1, the fourth circle: *T. foenum-graecum* vs MT039389.1, the fifth circle: *T. foenum-graecum* vs MT039390.1, the sixth circle: *T. foenum-graecum* vs MT039391.1, the seventh circle: *T. foenum-graecum* vs MT039392.1); the black circles represent the GC content; the green circles represent the GC skew+ (G>C); the purple circles represent the GC skew- (G<C)

*T. foenum-graecum* and five other Leguminous species (*Trifolium pratense*, *Trifolium meduseum*, *Trifolium grandiflorum*, *Trifolium aureum*, *Medicago truncatula*) were subjected to synteny analysis to tentatively determine their affinities. The results showed that *T. foenum-graecum* was the most genetically similar to *Medicago truncatula*. Schematic diagrams of the covariance and mt structures of these six plants are shown in Figs. 6 and 7. Among them, *Trifolium meduseum* has the largest mt genome of 348,724 bp whereas *Medicago truncatula* has the smallest mitogenome of 271,618 bp. They all have GC content of about 45%, further indicating that the plant mt genomes is relatively conserved.

**Fig. 8:**
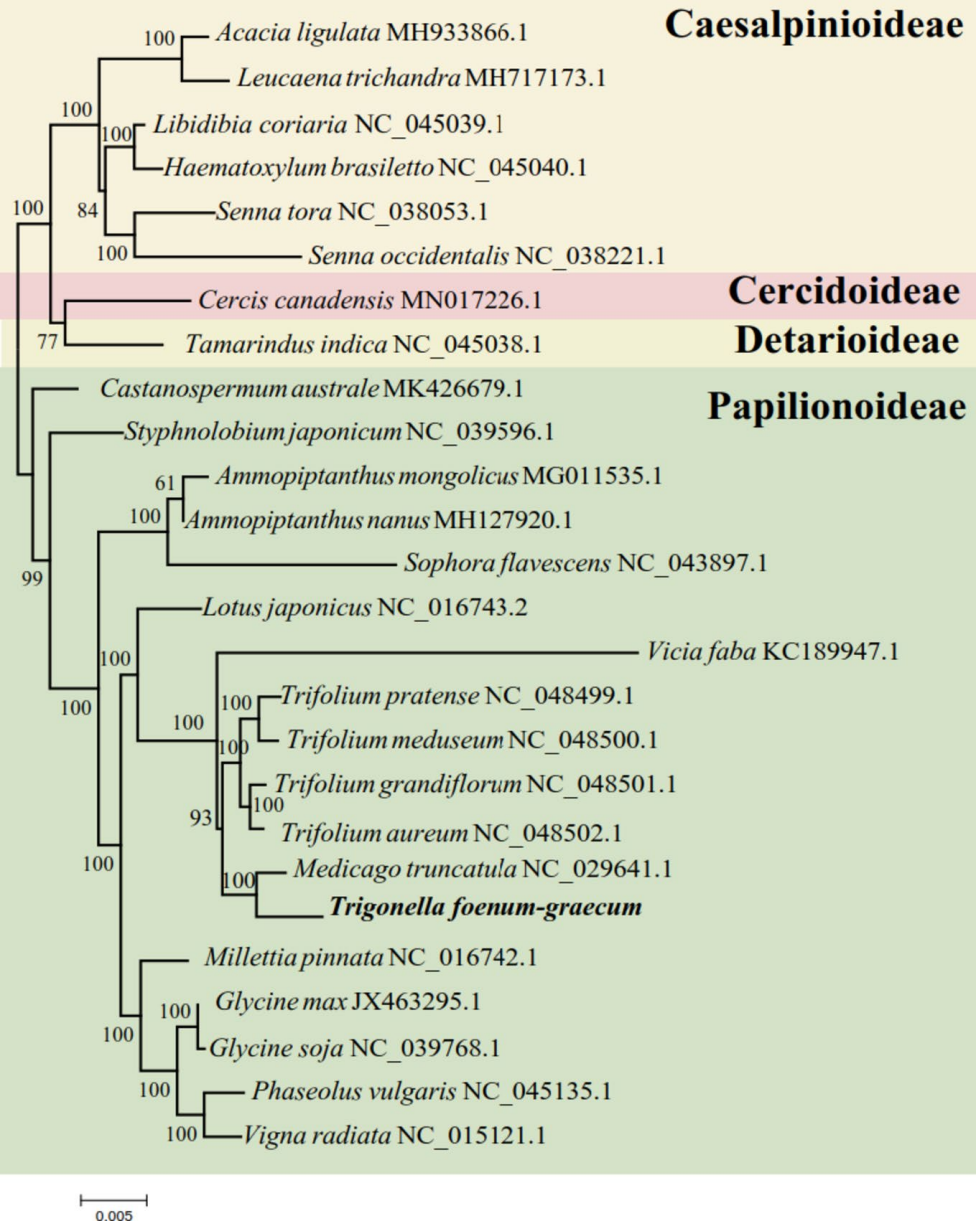
Phylogenetic relationships of *T. foenum-graecum* with 25 other plant species

*T. foenum-graecum* and 25 other Leguminosae species were subjected to phylogenetic analysis. In the comparison between *T. foenum-graecum* and other Papilionoideae plants, *T. foenum-graecum* is first linked to *Medicago truncatula* in a group with a maximum support of 100%. In a group connected with *Trifolium pratense*, *Trifolium meduseum*, *Trifolium grandiflorum*, and *Trifolium aureum*, the support is high at 93%. Caesalpinioideae, Cercidoideae and Detarioideae were compared as outgroups of the phylogenetic tree. The phylogenetic tree is shown in Fig. 8. There are 24 nodes in the phylogenetic tree, 18 of which have 100% support and 22 of which have more than 80% support.

### Substitution rates of PCGs

**Fig. 9:**
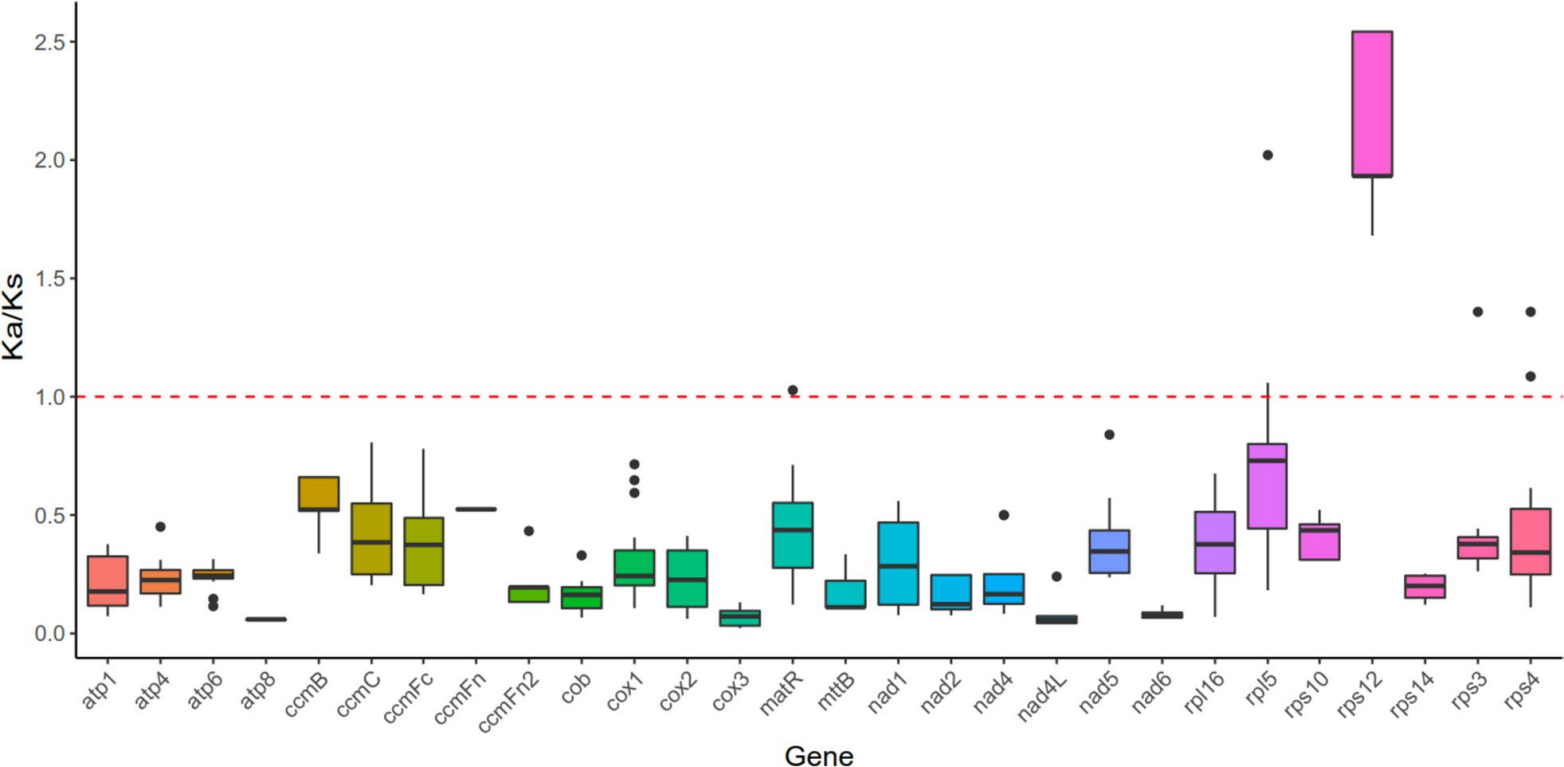
Boxplots of Ka/Ks between species. Ka/Ks values were obtained by aligning the CDS of genes of *T. foenum-graecum* with those of five plants (*Trifolium pratense*, *Trifolium meduseum*, *Trifolium grandiflorum*, *Trifolium aureum*, *Medicago truncatula*). The horizontal coordinates indicate the gene names and the vertical coordinates indicate the Ka/Ks values. In the box plots, the upper and lower edges of the rectangle indicate the upper and lower quartiles, the thick line inside the box indicates the median whereas the dots beyond the upper and lower edges of the box indicate outliers. Whiskers above and below the box indicate the range of Ka/Ks ratio values

The six Leguminosae plants (*T. foenum-graecum*, *Trifolium pratense*, *Trifolium meduseum*, *Trifolium grandiflorum*, *Trifolium aureum*, *Medicago truncatula*) were compared to analyze Ka/Ks values between species, as shown in Fig. 9. When Ka/Ks < 1, it indicates that these genes will continue to evolve under purifying selection; when Ka/Ks > 1, it indicates that positive selection of genes has occurred and new versions (alleles) of sequences have been preferred; when Ka/Ks = 1, it indicates that there is neutral selection [24]. Among the 28 PCGs counted, 23 genes (*atp1*, *atp4*, *atp6*, *atp8*, *ccmB*, *ccmC*, *ccmFC*, *ccmFn*, *ccmFn2*, *cob*, *cox1*, *cox2*, *cox3*, *mttB*, *nad1*, *nad2*, *nad4*, *nad4L*, *nad5*, *nad6*, *rpl16*, *rps10*, *rps14*) had Ka/Ks < 1, whereas in the case of the remaining five genes (*matR*, *rpl5*, *rps4*, *rps3*, *rps12*) Ka/Ks>1 was observed, i.e. for *matR* sequence between *Trifolium pratense* and *Trifolium meduseum*, for *rpl5* sequence when *T. foenum-graecum* vs *Trifolium pratense* and *Medicago truncatula* vs *Trifolium pratense* were compared, for *rps4* sequence in case when *Medicago truncatula* vs. *Trifolium grandiflorum* and *Trifolium meduseum* vs *Trifolium grandiflorum* were compared, and for *rps3* sequence between *Trifolium meduseum* and *Trifolium aureum*. The average Ka/Ks values for the *matR*, *rpl5*, *rps3* and *rps4* genes were 0.444, 0.726, 0.422, 0.464, respectively. In the case of *rps12* gene Ka/Ks > 1 were observed for 9 species combinations in which the gene sequence was compared, i.e. between *T. foenum-graecum* and remaining five analyzed species (*Trifolium pratense*, *Trifolium meduseum*, *Trifolium grandiflorum*, *Trifolium aureum*, *Medicago truncatula*) and between *Medicago truncatula* and four *Trifolium* species (*Trifolium pratense*, *Trifolium meduseum*, *Trifolium grandiflorum*, *Trifolium aureum*). The average Ka/Ks values for the *rps12* gene was 2.176.

### Chloroplast and mitochondrial homologous sequences

**Fig. 10:**
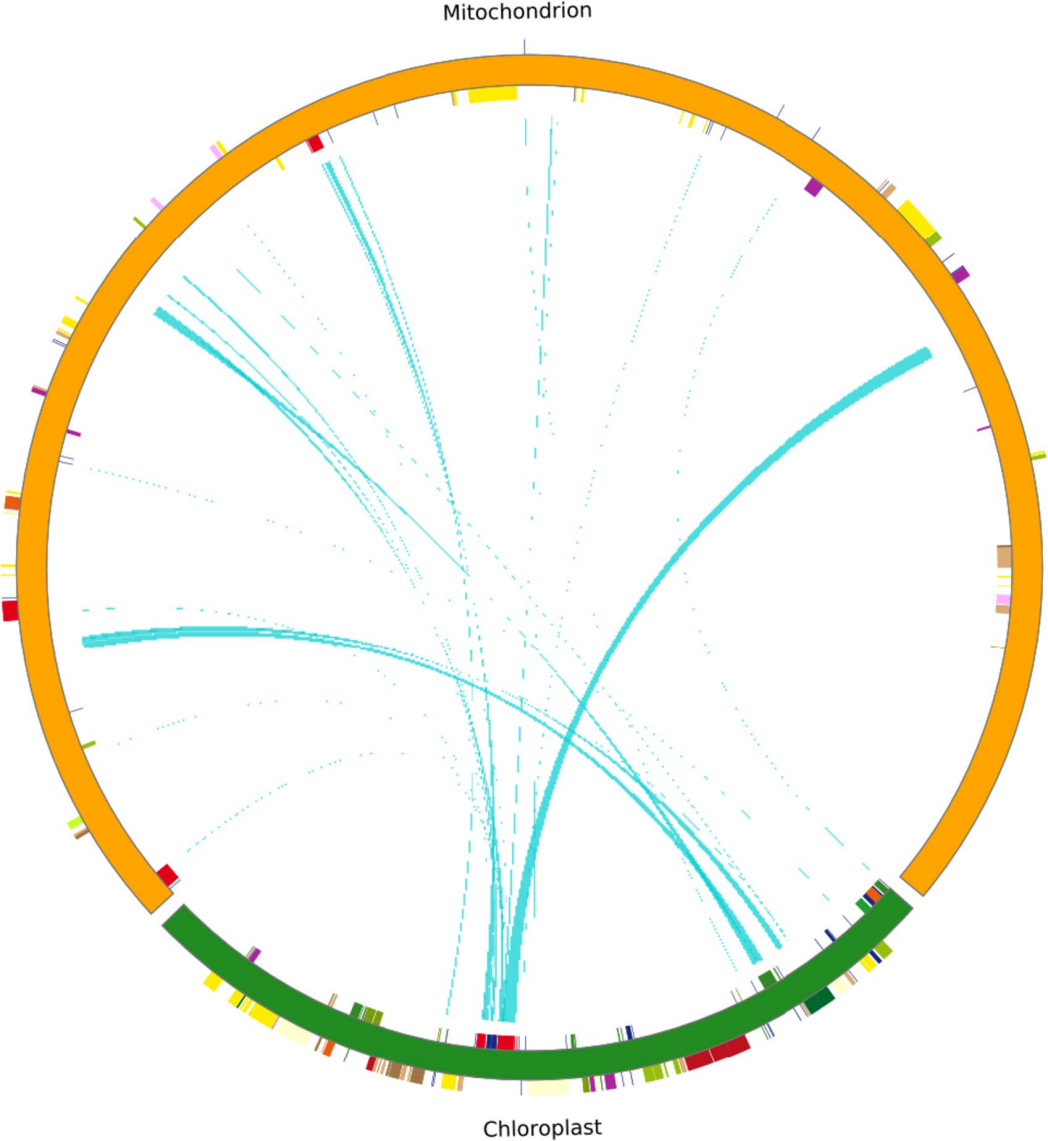
Distribution of homologous fragments in the cp genome and mitogenome of *Trigonella foenum-graecum*. The green segment of the circle represents the *T. foenum-graecum* cp genome, and the yellow segment represents the mt genome. Genes from the same complex are labeled with the same color, and the middle line connection indicates homologous sequences

Both the mt and cp genome in *T. foenum-graecum* were sequenced using the same tissue sample (leaf). Homology analysis revealed a transfer of DNA sequences from the cp genome to the mt genome. The mt and cp of *T. foenum-graecum* contain a total of 23 shared fragments, ranging from 35 to 2427 bp in length, for a total length of 10,023 bp, or 2.9% of the total genome length, as shown in Table 6. The analysis of homologous fragments of cp and mt sequences is shown in Fig. 10.

**Table 6:** Shared sequences of mt and cp of *T. foenum-graecum*

	Identity%	Length	Mismatches	Gap openings	gene
1	100	2427	0	0	*rrn4.5*(partical:6.73%) *rrn23*(partical:82.89%)
2	99.916	1188	1	0	*psbC*(partical:83.54%)
3	100	1140	0	0	*psaB*(partical:51.70%)
4	100	1016	0	0	*psbC*(partical:10.34%) *psbD*(partical:86.82%)
5	99.741	772	1	1	*rrn23*(partical:11.68%) *trnA-UGC*(partical:30.86%)
6	100	426	0	0	*trnI-GAU*(partical:55.72%)
7	92.708	384	4	5	*rrn23*(partical:13.68%)
8	99.288	281	2	0	*trnI-GAU*(partical:34.17%)
9	98.252	286	4	1	*trnW-CCA*; *petG*(partical:10.53%)
10	74.972	887	172	38	*rrn16*(partical:57.98%)
11	87.547	265	30	3	*rrn16*(partical:17.69%)
12	99.167	120	1	0	*psaA*(partical:5.27%)
13	91.27	126	11	0	*psaA*(partical:5.53%)
14	98.824	85	0	1	*trnN-GUU*
15	96.429	84	3	0	*trnD-GUC*
16	96.154	78	3	0	*trnH-GUG*
17	93.59	78	5	0	*trnM-CAU*
18	98.077	52	1	0	*rrn16*(partical:3.49%)
19	96.296	54	0	2	*ycf2*(partical:0.85%)
20	80.412	97	19	0	*rrn23*(partical:3.47%)
21	80.412	97	19	0	*rrn23*(partical:3.47%)
22	95.556	45	2	0	*rrn23*(partical:1.61%)
23	97.143	35	1	0	*rrn23*(partical:1.25%)

Percentage(%): The proportion of the length of the gene located in the homologous segment to the total length of the gene

## Discussion

Mitochondria are double-membrane organelles commonly found in eukaryotes and play an important role in life activities. Plant mt genomes exhibit complex and relatively conserved properties [11, 12], which create conditions for providing useful information for evolutionary and phylogenetic studies [25]. In recent years, with the continuous development of sequencing technology, the plant mt genomes has been studied more deeply.

In this study, second- and third-generation sequencing methods were used to study the mt genome of *T. foenum-graecum*. The mt genome of *T. foenum-graecum* was determined to have a circular structure, which is consistent with the previous reports on the structure of the many mitochondrial genomes of plants [26]. The variation in intron distribution can be a unique feature for certain species, and thus could be used to discriminate (potentially) the species [27]. A total of 11 genes in the mt genome of *T. foenum-graecum* contain one or more introns, which may play an important role in gene expression regulation [28]. GC content is an important factor in the characterization of species [29]. *T. foenum-graecum* mt genome GC content is 45.28%, which is similar to *Trifolium pratense* (NC_048499.1) 45.20%, *Trifolium meduseum* (NC_048500.1) 44.99%, *Trifolium grandiflorum* (NC_048501.1) 45.09%, *Trifolium aureum* (NC_048502.1) 44.88% and *Medicago truncatula* (NC_029641.1) 45.39%.

It has been suggested that the origin of the RNA editing sites is to repair mutations produced by themselves and UV irradiation during the evolution of plants [30–32]. There are significant differences in the role of RNA editing in the coding and non-coding regions of genes. RNA editing on the coding region of a gene often occurs in the first 2 bases of the codon, which can change the hydrophilicity and hydrophobicity of amino acids and ultimately affect the function of the protein [33, 34]. And RNA editing on non-coding regions plays an important role in mRNA splicing [35]. A total of 465 RNA editing sites were predicted in the 33 PCGs of the *T. foenum-graecum* mt genome, and all RNA editing sites were of the C-T editing type. The C-T editing type is the most common type of editing in plant mt genomes [36, 37], and the results of the study are the same as those previously reported.

Codon preference refers to favor to use one or more fixed codons in a given species or gene [38, 39]. When RSCU> 1, it indicates that the codon is used more frequently than other synonymous codons, which means that the codon generates bias; when RSCU = 1, all codons are used with the same frequency and the codon is unbiased [40]. When RSCU< 1 means that some codons are less abundant. A total of 32 codons were found to be biased in the *T. foenum-graecum* mt genome and these codons used a higher number of A/T bases.

Plant mitochondria are distinguished from chloroplasts by possessing numerous dispersed repetitive sequences. The variations in size and organization of plant mitochondrial genomes can largely be attributed to the presence and arrangement of these repetitive sequences, which significantly contribute to the evolutionary process of plant mitochondria [41]. In the current study, only two types (F and P) of scattered repeat sequences were identified in the mitochondrial genome of *T. foenum-graecum*, which is consistent with the findings in *Bupleurum chinense* [18]. The majority of SSRs were composed of adenine (A) and thymine (T) bases. Due to the two hydrogen bonds connecting A and T bases, the energy required to disrupt these bonds is lower compared to the guanine (G) and cytosine (C) bond. As a result, it is easier to induce changes in the A-T base pairs [18]. Furthermore, a total of 19 tandem repeats were detected, among which 12 showed a perfect match rate of 100%.

Pi reveals the magnitude of variation in nucleic acid sequences of different species [42]. In the *T. foenum-graecum* mt genome Pi values estimated for five genes (*rps12*, *rps3*, *rpl5*, *cox2*, and *atp6*) are relatively high, all greater than 0.01, indicating a high degree of variability. The regions where these genes are located could provide potential molecular markers for Leguminosae plant genetics [42]. In addition, synteny and phylogenetic studies revealed that *T. foenum-graecum* share high genetic similarity with the other five Leguminosae species (*Trifolium pratense*, *Trifolium meduseum*, *Trifolium grandflorum*, *Trifolium aureum* and *Medicago truncatula*), indicating that the process of mt genome evolution in Leguminosae is characterized by relative conservatism [12].

The ratio of Ka/Ks can determine the type of selection on genes and is important for reconstructing phylogenies and understanding the evolutionary dynamics of protein-coding sequences in closely related species [43]. In this study, 23 genes had Ka/Ks < 1, indicating that the genes were well conserved and will continue to evolve under purifying selective pressure. Five genes (*matR*, *rpl5*, *rps4*, *rps3*, *rps12*) had Ka/Ks > 1. It was found that the fastest evolving *Ajuga* gene, *rps12*, has lost all ancestral RNA editing sites mostly by C to T substitutions at the DNA level [44]. In the comparative analysis of *Artemisia giraldii* plasmid and mitochondrial genome, *rps12* also underwent positive selection [45]. A reverse transcriptase activity has been detected in potato mitochondria and it is easy to speculate that this activity is encoded by *matR* ORF [46]. *rpl5* regulates alternative splicing of transcription factor IIIA transcripts by binding to a conserved 5S rRNA-mimic structure that resides in an intron in the pre-mRNA [47]. The loss of editing at *ccmB*-43 and *rps4*–335 affects the maturation of cytochrome c and impairs the biogenesis of mitochondrial respiratory complexes, particularly complex III [48]. The U-to-C type editing amends numerous genomic stop codons in the *Adiantum capillus-veneris rps19*, *rps3* and *rpl16* sequences, thus, assuring the synthesis of complete and functional polypeptides [49]. This suggests that these five genes are important for plant mitochondria. They need to evolve to adapt to changes in its environment. PCGs with Ka/Ks > 1 are also present in other plants, and high gene Ka/Ks ratios play an important role in further studies of gene selection and evolution in species [50]. Migration of DNA sequences is frequently observed in the mt genome of plants [51]. Length and sequence similarity of migrating fragments vary between species [52]. In this study, 23 homologous fragments between the mt and cp genome of *T. foenum-graecum* were found. Of these, *psaA*, *psaB*, *psbC*, *psbD*, *petG* are present in both the mt and cp genomes and are associated with photosynthesis. These genes tend to be complete on the chloroplast genome, suggesting that these gene fragments may be migrating from cp to mt.

## Conclusions

In this study, the *T. foenum-graecum* mt genome was sequenced, assembled and annotated, and the DNA sequences of the annotated genes were analyzed. The *T. foenum-graecum* mt genome is 345,604 bp in length with 45.28% GC content. There are 59 genes, including 33 protein-coding genes, 21 tRNA genes, 4 rRNA genes, and 1 pseudo gene. Specific analyses of RNA editing sites, codon preference, three types of genomic repeats, nucleotide diversity, cp and mt homologous sequences were also performed. Synteny and phylogenetic analysis revealed that *T. foenum-graecum* had the highest genetic relationship with *Medicago truncatula*. Ka/Ks analysis revealed that most PCGs would continue to evolve under purifying selection pressure. In summary, this study has reported the complete sequence of the mt genome of *T. foenum-graecum* and provided its basic characteristics, which lays a foundation for further in-depth studies of the genus *Trigonella* (Leguminosae).

## Materials and methods

### Plant materials and DNA sequencing

*T. foenum-graecum* was cultivated at the medicinal herb planting base of the College of Pharmacy, Qinghai Minzu University (Xining, Qinghai, China). Young leaves from *T. foenum-graecum* seedlings were collected and cleaned with 70% alcohol to remove dust and soil. Then leaves were frozen in liquid nitrogen, and placed in pre-chilled 50 ml sealed bags. DNA of *T. foenum-graecum* was extracted and sequenced using Illumina Novaseq 6000 and Oxford Nanopore PromethION platforms for second- and third-generation sequencing, respectively. This sequencing was technically supported by GENEPIONEER (Nanjing, China). Using fastp v0.20.0 (https://github.com/OpenGene/fastp, Accessed 30 October 2022) software, the raw reads quality control was performed to discard reads with an average quality value of less than Q5, and filtering out reads for which the number (N) was greater than 5 [18]. The third-generation sequencing data was filtered using Filtlong v0.2.1 (https://github.com/rrwick/Filtlong, Accessed 30 October 2022) software.

### Assembly and annotation of the mt genome

Using the Minimap2 v2.1 [53] software, the raw thrid-generation data were aligned to the reference gene sequences (plant mitochondrial core genes) (https://github.com/xul962464/plant_mt_ref_gene, Accessed 30 October 2022), and the sequences with an alignment length greater than 50 bp were screened as candidate sequences on the alignment, from which the sequences with a larger number of aligned genes and a higher quality of the alignment were selected as the seed sequences. Then using Minimap2 v2.1 [53] to align the raw three-generation sequencing data to the seed sequences, screened the sequences with overlap greater than 1 kb and similarity greater than 70%, and added them to the seed sequences, and iteratively aligned the raw data to the seed sequences, so as to obtain all the three-generation sequencing data of the mt genome.

The resulting thrid-generation data were then corrected using the thrid-generation assembly software Canu v2.2 [54], the second-generation data were aligned to the corrected sequence using Bowtie2 v2.3.5.1 [55], and the aligned second-generation data and the corrected third-generation data were spliced together using the default parameters of Unicycler v0.4.8 (https://github.com/rrwick/Unicycle, Accessed 30 October 2022). Due to the complex physical structure of the mt genome, at this point, the corrected thrid-generation data were aligned to the contig obtained in the second step of Unicycler v0.4.8 using Minimap2 v2.1 [53] and manually determined the branching direction to obtain the final assembly results.

The encoded proteins and rRNAs were aligned to published and used as reference plant mt sequences (https://github.com/xul962464/plant_mt_ref_gene, Accessed 30 October 2022) using BLAST v2.6 (https://blast.ncbi.nlm.nih.gov/Blast.cgi, Accessed 30 October 2022). The tRNA was annotated using tRNAscanSE v2.0 [56] (http://lowelab.ucsc.edu/tRNAscan-SE/, Accessed 30 October 2022). ORFs were annotated using Open Reading Frame Finder (http://www.ncbi.nlm.nih.gov/gorf/gorf.html, Accessed 30 October 2022), with setting the minimum length to 102 bp to exclude redundant sequences and sequences that overlap with known genes, and sequences longer than 300 were annotated against the non-redundant protein sequences (nr) database. The final annotations were checked and manually adjusted if necessary. The Chloroplot [57] software (https://irscope.shinyapps.io/Chloroplot/, Accessed 30 October 2022) was used to create a mt genome map.

### Analysis of RNA editing sites and codon preference

RNA editing sites were analyzed using the Plant Predictive RNA Editor (PREP) suite [58]. RSCU is calculated as: actual frequency of use of the codon/theoretical frequency of use of the codon. We screened unique coding sequence (CDS) and calculated their RSCU values using self-encoded Perl script.

### Analysis of repeat sequences

Dispersed repeat sequences were identified using BLASTN v2.10.1 (https://blast.ncbi.nlm.nih.gov/Blast.cgi?PROGRAM=blastn&PAGE_TYPE=BlastSearch&LINK_LOC=blasthome, Accessed 23 November 2022) (parameters: - word_size 7, E-value 1e-5, remove redundancy, remove tandem duplicates) software. Tandem repeat sequences were identified using TRF v4.09 (https://github.com/Benson-Genomics-Lab/TRF, Accessed 23 November 2022) (parameters: 2 7 7 80 10 502,000 -f -d -m) software. SSRs were identified using MISA v1.0 (http://pgrc.ipk-gatersleben.de/misa/misa.html, Accessed 23 November 2022) (parameters: 1–10 2–5 3–4 4–3 5–3 6–3) [59] software. Circos v0.69–5 [60] (http://circos.ca/software/download/, Accessed 23 November 2022) was used to visualize and analyze the repeated sequences.

### Nucleotide diversity analysis

Multiple alignment of homologous gene sequences of *T. foenum-graecum* and five other species (*Trifolium pratense*, *Trifolium meduseum*, *Trifolium grandiflorum*, *Trifolium aureum*, *Medicago truncatula*) were performed using MAFFT v7.427 [61] software (−auto mode) and Pi values were calculated for each gene using DnaSP v5 [62].

### Comparative analysis of the mt genome structure

Comparative analysis of mt genome structure for closely related species (*Trifolium pratense*, *Trifolium meduseum*, *Trifolium grandiflorum*, *Trifolium aureum*, *Medicago truncatula*) was performed using the software CGView [63] with default parameters.

### Synteny analysis

Using the BLASTN v2.10.1 (parameters: - word_size 7, E-value 1e-5) software, fragments with a comparative length greater than 300 bp were screened, and the assembled species and the selected species were aligned sequentially to plot the covariance.

### Phylogenetic analysis

The complete mitochondrial genome sequences of species representing the order Leguminosae (Fabales) were downloaded from GenBank (Table 7). Maximum likelihood evolutionary tree was constructed using the 28 shared coding sequence (CDS) sequences extracted from these genomes. The sequences from different species were aligned using the MAFFT v7.427 [61] software, using the -auto mode. The aligned sequences were then concatenated and trimmed using trimAl v1.4.rev15, with a threshold of 0.7. After trimming, the best-fit model for the data was determined using jModelTest v2.1.10 [64] software, which identified the GTR model as the most appropriate. Subsequently, the maximum likelihood evolutionary tree was constructed using RAxML v8.2.10 [65] software with the following parameter settings: selected the GTRGAMMA model and set the bootstrap to 1000 replications.

**Table 7:** The name of the plant and the number of CDS were constructed for the phylogenetic tree

Specie names	Accession numbers of sequences	Numbers of CDS
*Acacia ligulata*	MH933866.1	31
*Leucaena trichandra*	MH717173.1	29
*Libidibia coriaria*	NC_045039.1	31
*Haematoxylum brasiletto*	NC_045040.1	31
*Senna tora*	NC_038053.1	31
*Senna occidentalis*	NC_038221.1	31
*Cercis canadensis*	MN017226.1	31
*Tamarindus indica*	NC_045038.1	31
*Castanospermum australe*	MK426679.1	31
*Styphnolobium japonicum*	NC_039596.1	31
*Ammopiptanthus mongolicus*	MG011535.1	29
*Ammopiptanthus nanus*	MH127920.1	31
*Sophora flavescens*	NC_043897.1	29
*Lotus japonicus*	NC_016743.2	31
*Vicia faba*	KC189947.1	31
*Trifolium pratense*	NC_048499.1	31
*Trifolium meduseum*	NC_048500.1	31
*Trifolium grandiflorum*	NC_048501.1	31
*Trifolium aureum*	NC_048502.1	31
*Medicago truncatula*	NC_029641.1	30
*Trigonella foenum-graecum*	OP605625	31
*Millettia pinnata*	NC_016742.1	31
*Glycine max*	JX463295.1	31
*Glycine soja*	NC_039768.1	30
*Phaseolus vulgaris*	NC_045135.1	30
*Vigna radiata*	NC_015121.1	30

### Ka/Ks values analysis

The gene sequences were aligned using MAFFT v7.310 [61] software. Ka/Ks values of genes were calculated using Ka/Ks Calculator v2.0 [66] software, and MLWL [67, 68] was chosen as the calculation method.

### Homologous sequence analysis

Homologous sequences between cp and mt genomes were found using BLAST v2.6 software, setting the similarity to 70% and the E-value to 1e-5. Mapping of the homologous fragments was performed using Circos v0.69–5 [60].

## Data Availability

Annotated sequences of *T. foenum-graecum* mt genome and cp genome were submitted to the NCBI under the following accession numbers OP605625 and OP747310, respectively.

## Abbreviations

*T. foenum-graecum*: *Trigonella foenum-graecum* L.
mt: Mitochondria
cp: Chloroplast
PCGs: Protein-coding genes
tRNA: Tranfer RNA
rRNA: Ribosomal RNA
SSRs: Simple sequence repeat
Pi: Nucleotide diversity
Ka/Ks: Nonsynonymous-to-synonymous substitution ratio
Met: Methionine
Lys: Lysine
Glu: Glutamate
Phe: Phenylalanine
Pro: Proline
Trp: Tryptophan
Gln: Glutamine
Gly: Glycine
Asp: Aspartate
Thr: threonine
Tyr: Tyrosine
Asn: Asparagine
Cys: Cysteine
His: Histidine
Leu: Leucine
RSCU: Relative synonymous codon usage
nr: non-redundant protein sequences
CDS: Coding sequence
